# Urachal Cyst, Meckel’s Diverticulum and Band, and Urachus

**DOI:** 10.21699/ajcr.v8i1.477

**Published:** 2017-01-05

**Authors:** Dileep Garg, Aditya Pratap Singh, Sunil Kothari, Ayush Kumar

**Affiliations:** 1 Department of Pediatric Surgery, SN Medical College Jodhpur, Rajasthan, India.; 2 Department of Pediatric Surgery, SMS Medical College Jaipur, Rajasthan, India.

**Dear Sir,**

A 4-year old boy presented with pain abdomen, bilious vomiting and constipation for three days. On examination, abdomen was distended and tender. X-ray abdomen revealed multiple air-fluid levels suggestive of intestinal obstruction. Ultrasonography abdomen revealed dilated bowel loops with minimal free fluid in peritoneal cavity. On exploration, there was a band causing obstruction of terminal ileum which was released. Band was arising from Meckel’s diverticulum which was connected to a cyst at umbilicus. On further exploration, the cyst was connected to urachus which was patent (Fig.1). There was no communication between bladder and urachus, cyst and urachus, Meckel's diverticulum and the cyst. Resection of Meckel’s diverticulum with ileum and end to end ileo-ileal anastomosis was done. Urachus and cyst were also excised. Patient recovered well postoperatively. Histopathology confirmed urachal cyst, urachus, and Meckel’s diverticulum.

Umbilicus is common passage for various structure during intrauterine life. These structures usually involute and their persistence result in a variety of anomalies at this site. Persistence of vitello-intestinal duct is common anomaly.[1] Urachal anomalies usually present as urachal sinus, patent urachus, or urachal cyst. Various authors have reported concurrent presence of both vitello-intestinal duct and urachal anomalies presenting with various features including intestinal obstruction and persistent discharge from the umbilicus.[2-5] Our case was unique as we found a Meckel’s diverticulum and a band which caused intestinal obstruction. Moreover, there was a urachal cyst at umbilicus which was attached to Meckel’s diverticulum on one side and to urinary bladder on other side through a patent tract which was not communicating with the urinary bladder. This was quite unusual.

**Figure:**
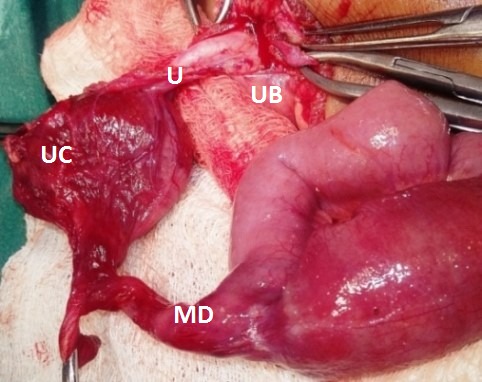
Figure 1: Showing urachal cyst (UC) attached to Meckel’s diverticulum (MD) on one side and urinary bladder (UB) on other side with a patent tract- urachus (U).

## Footnotes

**Source of Support:** Nil

**Conflict of Interest:** None declared

